# Prognostic Indices of Poor Nutritional Status and Their Impact on Prolonged Hospital Stay in a Greek University Hospital

**DOI:** 10.1155/2014/924270

**Published:** 2014-03-23

**Authors:** Georgia Tsaousi, Stavros Panidis, George Stavrou, John Tsouskas, Dimitrios Panagiotou, Katerina Kotzampassi

**Affiliations:** ^1^Department of Anesthesiology, Faculty of Medicine, Aristotle University of Thessaloniki, 54636 Thessaloniki, Greece; ^2^Department of Surgery, Faculty of Medicine, Aristotle University of Thessaloniki, 54636 Thessaloniki, Greece

## Abstract

*Background*. To ascertain the potential contributors to nutritional risk manifestation and to disclose the factors exerting a negative impact on hospital length of stay (LOS), by means of poor nutritional status, in a nonselected hospitalized population. *Materials and Methods*. NutritionDay project questionnaires were applied to 295 adult patients. Study parameters included anthropometric data, demographics, medical history, dietary-related factors, and self-perception of health status. Body Mass Index (BMI) and Malnutrition Universal Screening Tool (MUST) were calculated for each participant. MUST score was applied for malnutrition assessment, while hospital LOS constituted the outcome of interest. *Results*. Of the total cohort, 42.3% were at nutritional risk and 21.4% malnourished. Age, gender, BMI, MUST score, autonomy, health quality, appetite, quantity of food intake, weight loss, arm or calf perimeter (*P* < 0.001, for all), and dietary type (*P* < 0.01) affected nutritional status. Poor nutrition status (*P* = 0.000), deteriorated appetite (*P* = 0.000) or food intake (*P* = 0.025), limited autonomy (*P* = 0.013), artificial nutrition (*P* = 0.012), weight loss (*P* = 0.010), and arm circumference <21 cm (*P* = 0.007) were the most powerful predictors of hospital LOS >7 days. *Conclusion*. Nutritional status and nutrition-related parameters such as weight loss, quantity of food intake, appetite, arm circumference, dietary type, and extent of dependence confer considerable prognostic value regarding hospital LOS in acute care setting.

## 1. Introduction

Malnutrition is an ordinary clinical feature, with a currently estimated incidence ranging from 10 to 60% in acute hospital care, depending on the definition, clinical setting, and screening tool applied [1–9]. A notable proportion of hospitalized patients are not only at nutritional risk upon hospital admission, but their nutritional status deteriorates during their hospital stay as well [1, 5, 7]. Nutritional status derangement has a multifactorial origin, typically occurring during a continuity involving deficiency in dietary intake and/or increased requirements due to a disease state, from complications of an underlying disorder such as impaired absorption, excessive nutrient losses, and altered nutrient utilization or a combination of the aforementioned factors [2, 8, 10]. A physician confronts the challenge of weight loss, at several points of this continuity [8].

Impaired nutrition status has been identified as an independent predictor of depressed immune response, impaired wound healing, more frequent cardiac complications, higher readmission rate, and hence prolonged hospitalization or increased mortality, which eventually have an adverse secondary impact on health care facilities [1, 2, 5, 6, 11, 12].

Despite the progress in collective development of knowledge and compelling evidence, contemporary malnutrition rates have not altered significantly, as malnutrition in most clinical settings tends to be undetected and inappropriately addressed because it is not regarded as a high-priority entity [2–4, 6, 7, 10]. This has evolved as a matter of clinical concern, which can only be encountered through special attention to nutritional care of patients.

Identification of patients incurring notable risk of adverse events due to poor nutritional status is considered not only a core competency of nutrition practitioners, but is also indicated by clinical practice guidelines [7, 13, 14]. When devising strategies to deal with undernutrition and subsequently integrating them into everyday clinical routines, many factors have to be considered. Among them the influence of the disease per se on food intake and energy/nutrient requirements and the application of the more suitable tools for detecting risk or presence of nutritional deficiency are regarded as the most critical. Medical, psychological, and social causes have also to be considered. An optimal clinical assessment of nutrition status necessitates a detailed medical history, anthropometric measurements, laboratory tests, and dietary interviews, thus being a complex and time-consuming procedure [1, 3, 13].

Albeit, the screening of patients subjected to nutritional risk is considered as an essential initial step in the structured process of nutrition care, in order to identify those who will likely benefit from proper nutritional management, is usually overlooked, and is still away from being routinely implemented in hospitalized patients. On the occasion of the European NutritionDay project held in 2012 [15], we underwent a thorough nutritional status evaluation of nonselected adult patients in our university hospital, with a view to ascertain those at nutritional risk and the potential contributors to its manifestation. In addition, we got insight into factors, engendering negative outcomes such as prolongation of length of hospital stay (LOS), by means of poor nutritional status.

## 2. Experimental Methods

### 2.1. Study Population

This observational study evaluated the prospectively collected data of all heterogeneous adults hospitalized patients in different wards of a Greek university hospital upon the NutritionDay audit. Only patients of age 18 years and above were eligible for the study, as the criteria for defining malnutrition in patients younger than 18 years are complex and vary from the adult population [16].

Patients treated in an intensive care unit and those with deteriorated level of consciousness who had no caregivers or proxies to provide the necessary information were excluded. This study was conducted according to the guidelines laid down in the Declaration of Helsinki and all procedures involving human subjects/patients were approved by our hospital scientific council. Written informed consent was obtained from all subjects/patients, their families, or legal representatives.

### 2.2. Data Collection

Six groups of two designated health-care professionals each (nurses dieticians or doctors, one of whom worked in the patient's ward and the other independent) interviewed participants or their family members, where available, as well as reviewed consent patients' medical charts to fill out a standardized questionnaire prepared by European Society of Parental and Enteral Nutrition (ESPEN) organization for NutritionDay in Europe [15]. In particular, the following factors were registered: (1) anthropometric data (age, gender, measured weight, height, midarm, and calf circumference), (2) demographics (participating specialties and presurvey and total hospital LOS), (3) past and present medical history mainly involving a detailed list of reported comorbidities and the number of prescriptions received per day (as a surrogate marker for disease severity), (4) quality of health status assessed by the extent of dependence in everyday activities and participants' self-perceived adequacy of health status, and (5) dietary-related parameters (self-reported weight loss within the last 3 months, autonomy in everyday living, appetite status, and quantity and type of nutritional intake during hospitalization).

Body Mass Index (BMI) was calculated for all patients by a dietician or an experienced physician. BMI scores were then classified into four categories: BMI less than 18.5 is considered as underweight, BMI 18.5–24.9 as normal, BMI 25–30 as overweight, BMI 30 or greater as severely elevated (obese), and BMI more than 40 as extremely elevated (morbidly obese) [17].

The Malnutrition Universal Screening Tool (MUST) was calculated for all participants and was considered as the target variable for determination of the overall risk of malnutrition [18]. MUST is a simple screening tool, based on BMI, unintentional weight loss in the past 3 to 6 months, and acute disease effect or being unable to receive food for more than 5 days, for classifying subjects into one of the basic nutritional status categories as follows: 0 = low risk (well nourished), 1 = moderate risk, and 2 or more = high risk for malnutrition (malnourished). Patients were considered to be at high risk if they had a BMI <18.5 kg/m^2^ or experienced >10% unintentional weight loss in the previous 3–6 months or were nil by mouth for >5 days. Patients were considered to be at moderate risk if they had a BMI 18.5–20.0 kg/m^2^ or experienced 5–10% weight loss in the previous 3–6 months. All other patients were classified as low risk [18]. Total hospital LOS was recorded for every participant and constituted the outcome of interest, which was dichotomized to ≤7 and to >7 days.

A random check carried out by three experienced investigators was used to eliminate errors in the data collection on paper sheets and transferring processes before the data were entered into a computer database.

### 2.3. Statistical Analysis

One-way analysis of variance (ANOVA) was undertaken for comparison of means of continuous variables and normal distributed data, while a nonparametric rank test, the Kruskal-Wallis test, was used to compare means in the case of nonnormally and noncontinuously distributed data. Normality of data was assessed by Kolmogorov-Smirnov test. Categorical variables were assessed by a chi-square or Fisher's exact test when the expected value of a cell was less than 5. Odds ratios with 95% confidence intervals were computed using a univariate and multivariate stepwise logistic regression model with hospital LOS as the response variable. For all statistical procedures, a *P* value of less than 0.05 was considered significant. Data were analyzed using SPSS version 18.0 (SPSS Inc., Chicago, IL, USA).

## 3. Results

**Figure 1:**
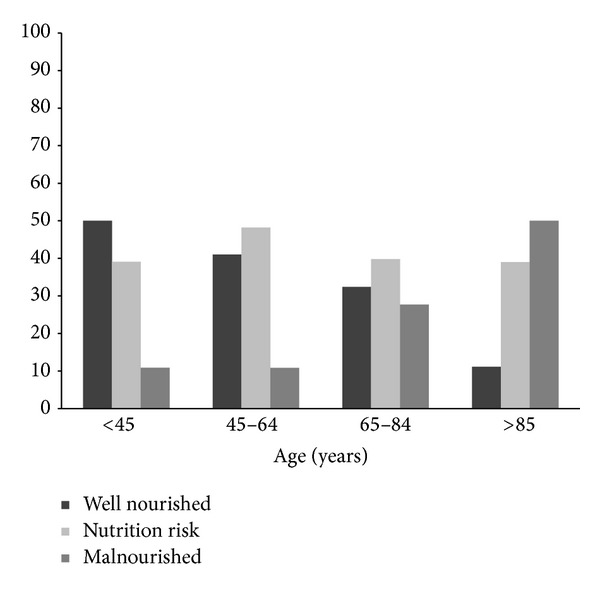
Nutrition status assessed by MUST classification related to age groups.* Note.* Data are expressed as percentage (%).

A total of 295 hospitalized patients were enrolled in the study.  Table 1 depicts the demographic characteristics, admission-related characteristics, and the studied parameters according to nutrition status of the participants. The mean age of our cohort was 63.6 (SD 17.2, range 18 to 100) years and the BMI was 26.7 (SD 5.1, range 14 to 42.6), while the male to female ratio was 1.5 : 1 (179/116). The age-dependent distribution of the MUST score differed significantly among the age subgroups (chi-square = 21.60, *P* < 0.001) as it is shown in Figure 1. In particular, nutritional status deterioration was proportional to advanced age. According to BMI subcategories 2.8% of cases (*n* = 8) were underweight, 39% (*n* = 115) with a normal BMI, 32% (*n* = 95) overweight, 24.8% (*n* = 73) obese, and 1.4% (*n* = 4) morbidly obese. The total hospital LOS of the study population was 7.4 (SD 7.5, range 1 to 65) days. Almost two out of three patients of the cohort had a hospital LOS less than 8 days (63.3%, *n* = 188).

Comorbidities were present in 167 (56.6%) patients, with the most common being hypertension (31.9%, *n* = 53), followed by diabetes mellitus (27.1%, *n* = 45), stroke (26.3%, *n* = 44), COPD (10.6%, *n* = 18), and cardiovascular diseases (4.1%, *n* = 7).

Weight loss more than 5% during the last 3 months and loss of appetite were recorded in 41% (*n* = 121) and in 30.8% (*n* = 91) of the patients, respectively. These two parameters were found to be significantly interrelated (chi-square = 20.68, *P* < 0.05). Quantity of received food less than normal during the last week was reported in 43.7% (*n* = 129) of the patients. Serious psychiatric disorders such as dementia or depression were observed in 8.1% (*n* = 24) of the study group and among them 95.8% (*n* = 23) were found to be at the risk of malnutrition or malnourished (chi-square = 36.01, *P* < 0.000). Furthermore, 36.3% (*n* = 107) of the patients reported that they were under stressful conditions during the last 3 months and the majority of them (90.6%, *n* = 97) were in abnormal nutrition status (chi-square = 61.78, *P* < 0.001).

Nutritional status was also influenced by quantity of food received per day. Among the patients received hospital food in the well-nourished (*n* = 104) and malnourished (*n* = 54) group, the majority in the first group (80%, *n* = 87) consumed two to three whole meals per day, while in the latter group 74.6% (*n* = 40) of the subjects consumed just one whole meal per day (*P* < 0.001).

**Figure 2:**
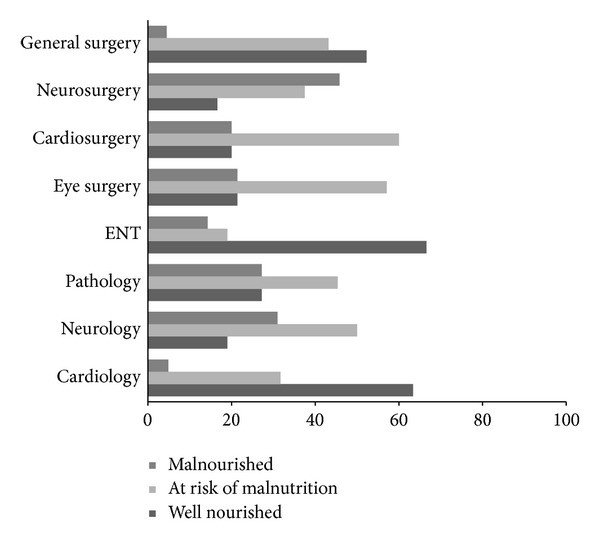
Distribution of all recruited patients per ward type stratified by nutritional status assessed by MUST classification.* Note*. Data are expressed as percentage (%). ENT: Eye-Nose-Throat.

Regular hospital food with no particular dietary plan was the source of nutrition for 58.6% (*n* = 173) of the patients; 34.6% (*n* = 102) of patients were given hospital food but modified for some form of special diet, and 6.8% (*n* = 20) were on enteral or total parenteral nutrition (TPN). The more adequate nutrition was recorded in patients receiving regular hospital food, while malnutrition was more frequent in patients under receiving artificial nutrition (enteral or TPN). According to the reason of admission, 42 (14.2%) patients had neurological problems, 99 (33.6%) pathological, and 113 (38.3%) surgical, while 41 (13.9%) patients presented with cardiac pathologies (chi-square = 25.61; *P* < 0.001). Nutritional status of the study population differed significantly (chi-square = 58.17; *P* = 0.000) according to the type of the ward being admitted (Figure 2). The best nutritional status presented the patients admitted to Eye-Nose-Throat (ENT) or to cardiology department (66.5% and 63.4%, resp.), followed by those admitted to general surgery department (52.3%). The higher prevalence of malnutrition risk was recorded in cardiosurgical and eye surgery cases (60% and 57.2%, resp.), while neurosurgery department had the higher incidence of severe malnourishment (45.8%). When the study population was classified into surgical and pathological subgroups, 27 (64.2%) of the malnourished subsets, 83 (63.8%) patients being at risk of malnutrition, and 72 (58.5%) of well-nourished patients involved pathological cases, but no significant difference in the nutritional status occurred in regard to surgical ones (chi-square = 0.89; *P* = 0.640). The length of hospital stay was comparable among the main subgroups according to the reason of admission. Neurological cases had higher length of hospital stay (8.1 ± 6.7 days; mean ± SD), while cardiological ones had lower (5.1 ± 4.2 days; mean ± SD).

Furthermore, the nutritional status was related to pressure ulcers development, in an important manner (chi-square = 34.48; *P* < 0.001). The total recorded incidence of pressure ulcers was 18.9% (*n* = 56), while the higher and lower incidence were observed in malnourished (66.8%, *n* = 28) and in well-nourished (6.6%, *n* = 8) patients, respectively.

When the variables tested as determinants of nutritional status were assessed by univariate logistic regression model as predictors of hospital LOS, BMI, nutrition risk assessed by MUST, quantity of food intake, appetite status, autonomy in everyday living, dietary type, recent weight loss, and arm or calf perimeter were identified as important determinants but not age and comorbidities (Table 2).

Variables that exhibited levels of statistical significance in the univariate analysis were entered into a stepwise logistic regression model, highlighting poor nutrition status (OR, 2.419; 95% CI, 1.523–3.841, *P* = 0.000), deteriorated appetite (OR, 3.681; 95% CI, 1.879–7.209, *P* = 0.000), quantity of food intake (OR, 1.409; 95% CI, 1.043–1.904, *P* = 0.025), limited autonomy (OR, 1.825; 95% CI, 1.135–2.936, *P* = 0.013), artificial diet (OR, 1.702; 95% CI, 1.122–2.583, *P* = 0.012), recent weight loss (OR, 3.903; 95% CI, 2.053–7.420, *P* = 0.010), and arm perimeter <21 cm (OR, 2.289; 95% CI, 1.259–4.163, *P* = 0.007), as the most powerful predictors of hospital LOS > 7 days.

## 4. Discussion

This study reports the association between anthropometric data, nutrition-related parameters, reason of admission, health status quality, and presurvey and total hospital LOS, with nutritional status adequacy, in acute care setting. Furthermore, malnutrition, impaired appetite, inadequate food intake, recent weight loss, reduced midarm perimeter, limited autonomy in everyday living, and artificial nutrition were identified as major contributors to prolonged hospital LOS.

Acute or chronic illness, injury, and social or environmental circumstances are notorious for engendering inflammatory, hypermetabolic, and/or hypercatabolic conditions. Traditionally, starvation-related malnutrition is attributed to inadequate protein and energy intake essentially over prolonged periods of time, due to reduced appetite and/or food intake, whereas disease-related malnutrition is a reaction to a disease state or to comorbidities, usually involving an inflammatory component [9, 19]. Individuals may exhibit a spectrum of clinical features ranging from “severe” malnutrition to mild or moderate malnutrition that, if left unrecognized and unaddressed, is likely to progress to a severely malnourished state [8].

A substantial body of evidence supports the considerable fluctuation in the prevalence of altered nutritional status among hospital populations, mainly attributed to the screening tools and elements applied, as well as the population and setting investigated [3, 4, 7, 11, 20, 21]. Albeit, malnutrition screening is recommended as the first step in nutritional care to allow early identification and treatment malnutrition; the optimum method for conducting nutritional assessment still remains controversial [3, 5, 7, 13, 22, 23]. A general comparison of the prevalence rate of malnutrition or risk for malnutrition development in our study population as it was assessed by MUST (21.4%) shows that it is within the reported range, further supporting the rather limited awareness on the issue of nutritional support in acute care setting [1–7, 18, 20, 23]. Body Mass Index (BMI) is a simple and objective measurement for determining the nutritional status and is an important component of several malnutrition screening tools [16, 22]. The differences in BMI scores across MUST categories were expected, as the BMI is a major item of this score. In a similar setting, Hiesmayr et al. [11] recorded a low BMI (<18%) in 6%, a normal BMI (18.5–25) in 40%, a moderately elevated BMI (25–30) in 30%, a severely elevated BMI (30–40) in 15%, and an extremely elevated BMI > 40 in 2% of the patients (9% were missing data). These findings are comparable to ours with the exception of the low BMI category, which included only 2.8% of our cases. The underestimation of malnutrition with BMI is in agreement with several reported data, further supporting the aspect that BMI could not serve as an accurate index of malnutrition [24–27]. For instance, a patient can have a high BMI and yet be undernourished if he has stopped eating because of an underlying disease. On the contrary, an individual could be thin with a low BMI but without malnutrition. Moreover, BMI was shown to be inaccurate in assessing a fatness risk factor for individuals [24].

Obtaining insight into predisposing factors for malnutrition should make it possible to identify subgroups of patients at risk [1, 13, 14, 28]. A well-documented risk factor for developing malnutrition is age [3, 4, 6, 23]. Higher age is associated with increased risk of malnutrition, as disease prevalence in this group increases and body composition changes. Sex, too, is another possible factor that could influence malnutrition as body composition changes occur differently in men and women in the various ageing phases, thus influencing the assessment and screening of malnutrition [29, 30]. In the literature, there is inconsistency regarding malnutrition prevalence in relation to gender [20, 29, 30]. In our analysis, the distribution of malnutrition according to gender differed significantly and affected women (60.3%) more than men (39.7%). The difference in mean age between malnourished and well-nourished patients is also clear (69.8 and 59.6 years, resp.), a profile that points to a heightened risk in aged females. However, this age effect highlighted in the univariate analysis disappeared when it was tested among the age groups. Although not statistically significant a tendency to malnutrition in patients aged over 65 years compared to younger ones could be detected. Probably in a larger group of patients this tendency could turn out to be significant.

Weight loss (both volitional and unintentional) is one of the key historical nutrition assessment indicators, which has been associated with long-term mortality in numerous studies [9, 11, 27–29]. Clinical significance is determined both by the degree and duration of weight loss [27]. A large cohort of 3122 participants demonstrated that poor food intake during hospitalization is a risk factor for malnutrition [29]. Our findings are in accordance with those of Hiesmayr et al. [11] who reported weight loss in the previous 3 months in 42% of participants, while eating less than normal during the previous week was self-reported by 51% of patients. In our study group the same parameters were 41% and 43.7%, respectively. Both BMI and weight loss constitute criteria involved in MUST estimation. We confirmed that both of these criteria can independently predict clinical outcome, varying by the clinical circumstance, but apparently when they are assessed together they are considered better predictors than each alone [18]. One of the main risk factors of undernourishment, especially in the hospital setting, is health status deterioration [3]. A diversity of disease-related aspects that decrease food intake even when food is available have been highlighted including loss of appetite, nausea, psychological problems, and difficulties in chewing, tasting, swallowing, and digestion. Furthermore, it has been demonstrated that nutrient requirements are promoted by disease, which indicates that even normal intake could be insufficient for such patients. Noteworthy, there is no universally accepted measure for disease severity in normal hospital ward patients [11]. We therefore used presurvey length of hospitalization, the presence of comorbidities, the extent of dependence, and health status self-assessment as indicators of health status adequacy. With the exception of comorbidities, all the aforementioned parameters were significantly related to nutritional status of the studied group, which is indicative of the close relationship among influence of the severity of illness and malnutrition. However, a trend towards receiving more drugs per day was shown in undernourished patients. Furthermore, severity of nutritional status impairment seems to be closely associated with the extent of an individual's dependence in everyday activities [30].

In the vast majority (93.2%) of our patients, normal by mouth nutrition was feasible, and more than half of them were able to ingest the regular hospital diet. These findings are in line with those of Imoberdorf et al. [4] and Pirlich et al. [20] who recorded normal by mouth nutrition in 86% and 82% of their subjects, respectively, while more than half of them were able to ingest the regular hospital diet.

Medical/surgical history and clinical diagnosis could raise suspicion for the possible existence of underlying inflammation and malnutrition. In our study population, the higher prevalence of malnutrition risk was recorded in cardiosurgical and neurological cases followed by pathological and ENT ones, while neurosurgery department presented the higher incidence of severe malnourishment. These findings could be interpreted under the light of the patients' profile being different in many specialties and the fact that nutrition status is highly dependent upon the degree of awareness among the caring physicians, which seems to be deescalated in highly specialized medical fields.

A great body of evidence demonstrates how clinical malnutrition negatively affects the recovery from a disease, trauma, or surgery, and it is generally associated with evident negative consequences in all aspects of hospital stay [20, 29, 31, 32]. Among the various complications arising from poor nutrition status, pressure ulcer development is regarded as one of the commonest with a reported incidence ranging from 0.4% to 38% in acute care facilities [33–35]. In our setting, pressure ulcers were used as a representative complication related to malnutrition and we verified that pressure ulcer presented in 18.9% of the patients and was associated with nutritional status in an important manner. Banks et al. [35] confirmed the significant association between impaired nutritional status and the presence of pressure ulcers with an increased odds risk of greater than two times for moderate malnutrition to almost five times for severe malnutrition in individuals in acute care facilities.

As has already been pointed out, there is documented evidence to suggest that malnourished patients incur greater hospitalization costs, related to longer LOS, and greater utilization of hospital resources [10, 20, 29, 32, 36–38]. The majority of the published data converge on prolongation of hospital LOS by an average of 2–5 days among malnourished and well-nourished patients [29, 32, 36, 37]. In our cohort, the mean hospital LOS for patients defined as malnourished was almost four times higher than those defined as well nourished (14.1 days compared to 3.7 days), which approximates the findings of Marco et al. [6] who reported a mean hospital LOS 18.1 days in undernourished hospitalized population as compared with 3.4 days for well-nourished ones.

Considering several both nonnutritional and nutritional factors that can influence LOS, it is important to account for these factors [38, 39]. From our univariate analysis, nonnutritional factors such as gender, extent of dependence in everyday activities and nutritional ones such as BMI, MUST score, midarm and calf circumference, weight loss, appetite status, quantity of food intake, and dietary type emerged as sensitive markers not only for undernutrition identification but for hospital LOS prediction as well. Among them, MUST score, recent weight loss, appetite, quantity of food consumed, dietary type, and autonomy were highlighted as the most powerful predictors of hospital LOS, in our general adult acute care population. These factors are modifiable, in contrast to other risk factors such as age or sex or disease severity and their evaluation is simple, inexpensive, and noninvasive.

Even though the negative impact of poor nutritional status upon morbidity is a well-established issue [10, 20, 23, 29, 32, 36, 37], limited documented data have controlled for other coexisting factors. Kandiah et al. [40] reported a positive association between extended LOS and greater plate waste. Furthermore, appetite deterioration could serve as an additional contributing factor for reduced food consumption, while limited autonomy reflects the health status deterioration, the possibility of undernutrition upon hospital nutrition, and the inability of proper access to food. In a similar clinical setting, Raslan et al. [23] showed that the patients who had lost a considerable amount (>10%) of their body weight in the 6 months before hospital admission presented prolonged length of hospital stay.

The authors acknowledge some limitations of the present study. Our single-center population not only is heterogeneous and incorporates patients from different age groups but it also involves a rather small sample. Moreover, as no gold standard tool has been developed for accurate and reliable nutritional status screening, MUST score may yield different sensitivity and specificity as an index of nutrition status assessment risk in diverse age categories and clinical settings [23].

In conclusion, the findings of the present study substantiate that poor nutritional status, recent weight loss, reduced quantity of food intake, impaired appetite, limited autonomy in everyday activities, and artificial nutrition confer noteworthy prognostic value regarding hospital LOS in acute care setting. The economic consequence of this health outcome index is substantial, as the LOS was four times longer for malnourished than for well-nourished patients. Our results accentuate the need for constant awareness of the medical care providers in Greek hospitals regarding the malnutrition issue and the importance of implementing simple nutrition screening tools for prompt identification of those patients at high risk of nutritional deficiency.

## Figures and Tables

**Table 1:** Participants' characteristics according to MUST categories.

Variable	Well nourished	At risk of malnutrition	Malnourished	*P* value
*N* (%)	107 (36.2)	125 (42.3)	63 (21.4)	
Age (years)	59.6 (17.7)	63.9 (16.2)	69.8 (16.3)***	0.001
Females	29 (27.1)	48 (38.4)	38 (60.3)	0.000
BMI	28.1 (4.8)	26.2 (4.7)*	25.4 (5.7)**	0.001
Presurvey LOS (days)	6.3 (7.1)	7.3 (8.7)	9.8 (8.2)*	0.017
Hospital LOS (days)	3.7 (2.3)	7.4 (4.6)***	14.1 (9.1)***	0.000
Comorbidities				
Hypertension	20 (18.7)	16 (12.8)	10 (15.8)	0.329
Diabetes mellitus	18 (16.9)	26 (20.8)	13 (20.6)	
Stroke	17 (15.8)	14 (11.2)	7 (11.2)	
COPD	7 (6.6)	6 (4.8)	5 (7.9)	
Cardiovascular disease	6 (5.6)	2 (1.6)	0	
No comorbidity	39 (36.4)	61 (48.8)	28 (44.5)	
Autonomy				
Out of home	100 (93.3)	88 (70.9)	19 (30.1)	0.000
Home only	5 (4.6)	20 (16.2)	13 (20.7)	
Bedridden	2 (2.1)	17 (12.9)	31 (49.2)	
Health status self-assessment				
Fine	6 (5.7)	4 (3.4)	1 (1.6)	0.000
Very good	20 (19.1)	20 (16.2)	3 (4.7)	
Good	38 (35.2)	31 (24.8)	11 (17.5)	
Satisfactory	32 (29.5)	33 (26.5)	17 (26.9)	
Poor	11 (10.5)	37 (29.1)	31 (49.3)	
Appetite status				
Abnormal	14 (13.1)	40 (31.6)	48 (75.9)	0.000
Normal	93 (87.3)	85 (68.2)	15 (24.1)	
Dietary type				
Regular diet	76 (71.1)	69 (55.2)	28 (44.4)	0.003
Specific diet	27 (25.2)	49 (39.2)	26 (41.2)	
Enteral nutrition	2 (1.9)	2 (1.7)	6 (9.5)	
TPN	2 (1.9)	5 (3.9)	3 (4.9)	
Quantity of food last week^§^				
Normal	78 (74.7)	69 (58.3)	17 (31.4)	0.000
Less than normal	17 (16.5)	17 (14.4)	8 (15.4)	
Half normal	7 (6.5)	18 (15.2)	8 (14.8)	
Quarter normal	2 (2.3)	14 (12.1)	21 (38.4)	
Recent weight loss				
Yes	29 (25.2)	57 (45.5)	46 (73.3)	0.000
No	64 (60.5)	50 (39.8)	6 (10)	
Gain weight	12 (11.8)	15 (12.3)	2 (3.3)	
Do not know	2 (2.5)	3 (2.4)	9 (14.7)	
Arm perimeter <22 cm	2 (1.9)	9 (7.2)	36 (57.1)	0.000
Calf perimeter <31 cm	7 (6.5)	28 (22.4)	45 (71.4)	0.000

*Note.* Data are expressed as mean (SD) or number of patients (%).

MUST: Malnutrition Universal Screening Tool; BMI: Body Mass Index; MNA: Mini Nutritional Assessment; LOS: length of stay; COPD: chronic obstructive pulmonary disease; TPN: total parenteral nutrition; *P < 0.05; **P < 0.01; ***P < 0.001 versus well-nourished group.

**Table 2:** Univariate logistic regression analysis of the association between factors related to nutrition status and prolongation of LOS.

Variable	OR (95% CI)	*P* value
Comorbidities	1.089 (0.674–1.760)	0.727
BMI	1.760 (1.281–2.419)	0.000
MUST		
Well nourished	1.0	Reference
Risk of malnutrition	2.054 (1.042–4.052)	0.038
Malnutrition	7.330 (3.494–15.376)	0.000
Recent weight loss	2.950 (1.1797–4.850)	0.000
Abnormal appetite status	1.745 (1.252–3.151)	0.000
Quantity of food last week		
Normal	1.0	Reference
50% of normal	1.577 (0.770–3.228)	0.213
25% of normal	4.318 (1.906–8.983)	0.000
None	7.483 (3.414–16.403)	0.000
Autonomy		
Walk with no assistance	1.0	Reference
Walk only with assistance	3.091 (1.515–6.303)	0.002
Bedridden	6.831 (3.105–5.028)	0.000
Nutrition type		
Hospital food	1.0	Reference
Specific diet	1.681 (1.004–2.815)	0.048
Enteral	4.109 (1.109–11.219)	0.007
TPN	6.560 (1.672–15.739)	0.005
Midarm circumference <21 cm	6.519 (2.291–18.549)	0.000
Calf circumference <31 cm	2.551 (1.506–4.318)	0.000
Health status self-assessment	1.572 (1.248–1.979)	0.000

*Note.* Univariate analysis with each variable entered in logistic regression analysis separately.

*N* = 188 with hospital LOS ≤ 7 days versus *N* = 107 with hospital LOS > 7 days.

The odds ratio (OR) for “BMI” is for each increase in score unit, whereas all other variables are categorical.

## References

[1] Schindler K, Pernicka E, Laviano A (2010). How nutritional risk is assessed and managed in European hospitals: a survey of 21,007 patients findings from the 2007-2008 cross-sectional nutritionDay survey. *Clinical Nutrition*.

[2] Korfali G, Gündoğdu H, Aydintuğ S (2009). Nutritional risk of hospitalized patients in Turkey. *Clinical Nutrition*.

[3] Meijers JMM, Halfens RJG, van Bokhorst-de van der Schueren MAE, Dassen T, Schols JMGA (2009). Malnutrition in Dutch health care: prevalence, prevention, treatment, and quality indicators. *Nutrition*.

[4] Imoberdorf R, Meier R, Krebs P (2010). Prevalence of undernutrition on admission to Swiss hospitals. *Clinical Nutrition*.

[5] Sorensen J, Kondrup J, Prokopowicz J (2008). EuroOOPS: an international, multicentre study to implement nutritional risk screening and evaluate clinical outcome. *Clinical Nutrition*.

[6] Marco J, Barba R, Zapatero A (2011). Prevalence of the notification of malnutrition in the departments of internal medicine and its prognostic implications. *Clinical Nutrition*.

[7] Rasmussen HH, Holst M, Kondrup J (2010). Measuring nutritional risk in hospitals. *Clinical Epidemiology*.

[8] White JV, Guenter P, Jensen G, Malone A, Schofield M (2012). Consensus statement: academy of nutrition and dietetics and American society for parenteral and enteral nutrition: characteristics recommended for the identification and documentation of adult malnutrition (undernutrition). *Journal of Parenteral and Enteral Nutrition*.

[9] Jensen GL, Mirtallo J, Compher C (2010). Adult starvation and disease-related malnutrition: a proposal for etiology-based diagnosis in the clinical practice setting from the International Consensus Guideline Committee. *Clinical Nutrition*.

[10] Barker LA, Gout BS, Crowe TC (2011). Hospital malnutrition: prevalence, identification and impact on patients and the healthcare system. *International Journal of Environmental Research and Public Health*.

[11] Hiesmayr M, Schindler K, Pernicka E (2009). Decreased food intake is a risk factor for mortality in hospitalised patients: the NutritionDay survey 2006. *Clinical Nutrition*.

[12] Mirmiran P, Hosseinpour-Niazi S, Hamayeli Mehrabani H, Kavian F, Azizi F (2011). Validity and reliability of a nutrition screening tool in hospitalized patients. *Nutrition*.

[13] Mueller C, Compher C, Ellen DM (2011). A.S.P.E.N. clinical guidelines: nutrition screening, assessment, and intervention in adults. *Journal of Parenteral and Enteral Nutrition*.

[14] Heyland DK, Dhaliwal R, Jiang X, Day AG (2011). Identifying critically ill patients who benefit the most from nutrition therapy: the development and initial validation of a novel risk assessment tool. *Critical Care*.

[15] http://www.nutritionday.org.

[16] Elia M (2000). Guidelines for detection and management of malnutrition. *Standing Committee of BAPEN*.

[17] Prospective Studies Collaboration PSC (2009). Body-mass index and cause-specific mortality in 900000 adults: collaborative analyses of 57 prospective studies. *The Lancet*.

[18] Stratton RJ, Hackston A, Longmore D (2004). Malnutrition in hospital outpatients and inpatients: prevalence, concurrent validity and ease of use of the “malnutrition universal screening tool” (“MUST”) for adults. *British Journal of Nutrition*.

[19] Muscaritoli M, Anker SD, Argilés J (2010). Consensus definition of sarcopenia, cachexia and pre-cachexia: joint document elaborated by Special Interest Groups (SIG) “cachexia-anorexia in chronic wasting diseases” and “nutrition in geriatrics”. *Clinical Nutrition*.

[20] Pirlich M, Schütz T, Norman K (2006). The German hospital malnutrition study. *Clinical Nutrition*.

[21] Gout BS, Barker LA, Crowe TC (2009). Malnutrition identification, diagnosis and dietetic referrals: are we doing a good enough job?. *Nutrition and Dietetics*.

[22] Anthony PS (2008). Nutrition screening tools for hospitalized patients. *Nutrition in Clinical Practice*.

[23] Raslan M, Gonzalez MC, Gonçalves Dias MC (2010). Comparison of nutritional risk screening tools for predicting clinical outcomes in hospitalized patients. *Nutrition*.

[24] Vandewoude M (2010). Nutritional assessment in geriatric cancer patients. *Supportive Care in Cancer*.

[25] Nourissat A, Mille D, Delaroche G (2007). Estimation of the risk for nutritional state degradation in patients with cancer: development of a screening tool based on results from a cross-sectional survey. *Annals of Oncology*.

[26] Kruizenga HM, Wierdsma NJ, van Bokhorst MAE (2003). Screening of nutritional status in the Netherlands. *Clinical Nutrition*.

[27] Zekry D, Herrmann FR, Vischer UM (2013). The association between the Body Mass Index and 4-year all-cause mortality in older hospitalized patients. *The Journals of Gerontology A*.

[28] Jensen GL, Wheeler D (2012). A new approach to defining and diagnosing malnutrition in adult critical illness. *Current Opinion in Critical Care*.

[29] Agarwal E, Ferguson M, Banks M (2013). Malnutrition and poor food intake are associated with prolonged hospital stay, frequent readmissions, and greater in-hospital mortality: results from the Nutrition Care Day Survey. *Clinical Nutrition*.

[30] Perissinotto E, Pisent C, Sergi G, Grigoletto F, Enzi G (2002). Anthropometric measurements in the elderly: age and gender differences. *British Journal of Nutrition*.

[31] Norman K, Pichard C, Lochs H, Pirlich M (2008). Prognostic impact of disease-related malnutrition. *Clinical Nutrition*.

[32] Braunschweig C, Gomez S, Sheean PM (2000). Impact of declines in nutritional status on outcomes in adult patients hospitalized for more than 7 days. *Journal of the American Dietetic Association*.

[33] Fisher AR, Wells G, Harrison MB (2004). Factors associated with pressure ulcers in adults in acute care hospitals. *Advances in skin & wound care*.

[34] Schoonhoven L, Grobbee DE, Donders ART (2006). Prediction of pressure ulcer development in hospitalized patients: a tool for risk assessment. *Quality and Safety in Health Care*.

[35] Banks M, Bauer J, Graves N, Ash S (2010). Malnutrition and pressure ulcer risk in adults in Australian health care facilities. *Nutrition*.

[36] Lim SL, Ong KCB, Chan YH, Loke WC, Ferguson M, Daniels L (2012). Malnutrition and its impact on cost of hospitalization, length of stay, readmission and 3-year mortality. *Clinical Nutrition*.

[37] Correia MITD, Waitzberg DL (2003). The impact of malnutrition on morbidity, mortality, length of hospital stay and costs evaluated through a multivariate model analysis. *Clinical Nutrition*.

[38] Liu Y, Phillips M, Codde J (2001). Factors influencing patients’ length of stay. *Australian Health Review*.

[39] Raslan M, Gonzalez MC, Torrinhas RSMM, Ravacci GR, Pereira JCR, Waitzberg DL (2011). Complementarity of Subjective Global Assessment (SGA) and Nutritional Risk Screening 2002 (NRS 2002) for predicting poor clinical outcomes in hospitalized patients. *Clinical Nutrition*.

[40] Kandiah J, Stinnett L, Lutton D (2006). Visual plate waste in hospitalized patients: length of stay and diet order. *Journal of the American Dietetic Association*.

